# Sensor-based human activity recognition using wrist and ankle wearables

**DOI:** 10.3389/fbioe.2026.1926076

**Published:** 2026-09-21

**Authors:** Ojan Majidzadeh Gorjani, Rene Jaros, Petr Bilik

**Affiliations:** Department of Cybernetics and Biomedical Engineering, Faculty of Electrical Engineering and Computer Science, VSB–Technical University of Ostrava, Ostrava, Czechia

**Keywords:** activity recognition, artificial neural network, classification, machine learning, smart homes

## Abstract

**Introduction:**

This article proposes using dual-body sensor placement on the right ankle and wrist with raw multi-sensor fusion (accelerometer, magnetometer, and gyroscope at 66.6 Hz) to capture complementary upper- and lower-body kinematics across nine complex daily activities and real-time integration with smart homes.

**Methods:**

Two independent datasets were obtained from participants performing different activities each at intervals of 5–10 min and with real-time labeling. Classification was carried out using six different machine learning architectures (SVM, Logistic Regression, ANN, C&RT, Random Forest, and C5.0).

**Results:**

Random Forest and Multilayer Perceptron (ANN) models demonstrate superior cross-dataset generalization, achieving 93.48% accuracy when evaluated on fully independent sessions. Predictor importance analysis reveals distinct kinematic dependencies, where leg magnetometers dominate elevation-based activities (stair climbing) and wrist accelerometers drive fine-motor actions (typing, writing). Integrating a 120-sample majority-voting filter effectively eliminates transient classification noise without compromising real-time responsiveness (< 2s latency). Finally, the framework is validated via C#, PMML extraction, and Falcon.NET integration for real-time KNX smart home control.

**Discussion:**

The study findings highlight the feasibility of integrating wearable HAR systems into smart homes for real-time monitoring and adaptive assistance.

## 1 Introduction

Human activity recognition (HAR) in smart homes is an expanding research field due to its importance in improving comfort, safety, and assistance for populations such as the elderly or individuals with mobility limitations. Real-time monitoring enables automated detection of daily activities, early detection of abnormal activity, potentially dangerous situations, and provides context-aware services. Typically, this involves discerning and identifying human actions through the analysis of camera footage or data from wearable sensors. With the advancements of mobile and wearable technologies, sensor-based HAR has become increasingly accessible and practical. This is particularly advantageous given that camera-based alternatives frequently raise privacy concerns and face limitations in terms of coverage. Despite advances in HAR using wearable sensors and smart devices, many existing studies focus on limited sensor placements or restricted activity sets, rely on single-sensor placements or focus on limited activity sets, which can lead to misclassification, reduced robustness, and limited applicability for real-world monitoring. Using data from both the ankle and the wrist, this study aims to capture complementary upper and lower body movements, improving the classification accuracy in a few selected daily activities that can be applied to independent living, health monitoring and adaptive smart home environments, supporting populations that benefit the most from the most continuous and reliable activity tracking.

HAR systems often uses Artificial Neural Networks (ANN) and deep learning, particularly when processing sensory data. Researchers have actively explored these techniques. For example, Chen et al. (2022) took a deep dive into applying deep learning to sensor-based HAR. Similarly, Khan and Ahmad (2021) introduced an attention-based multihead model, employing one-dimensional convolutional neural networks in each “head” to extract features from sensor data. Gao et al. (2021a) developed a novel multibranch convolutional neural network that uses a selective kernel mechanism, leading to improved HAR accuracy without significantly increasing computational demands. Others, like Gil-Martín et al. (2021), have systematically evaluated various CNN-based strategies for HAR. To enhance the representation of spatio-temporal features and boost system performance, Basly et al. (2022) proposed a deep temporal residual system for recognizing daily living activities. Furthermore, Islam and Iqbal (2021) presented a hierarchical multimodal representation learning approach for HAR, built on graphical attention mechanisms. Gao et al. (2021b) also contributed a dual attention method, combining channel and temporal attention in residual networks to improve feature representation for sensor-based HAR tasks. In a move toward end-to-end learning, Prabono et al. (2021) presented a hybrid deep architecture for sensor-based HAR with domain adaptation. Even smartphone data are being used, as shown by Suriani and Rashid (2021), who used an open dataset of accelerometer data. In particular, these approaches often involve the conversion of analog input data into spike trains using rate-based encoding.

Sensor-based Human Activity Recognition (HAR) has had many advances, but single-sensor setups like just using a smartphone or smartwatch which struggled to tell complex, similar movements apart. They lack the spatial detail needed to differentiate actions like vacuuming versus walking, or typing versus writing, leading to major overlap in the data. On the flip side, multi-sensor systems usually depend on heavy feature engineering or slow, complex models that introduce too much latency for real-time smart home automation. Many HAR studies test their models using standard random k-fold cross-validation on data from a single session. This inflates the accuracy numbers and does not reflect how the system performs over time across different sessions. To fix this, we need a practical, low-latency system using two wearable sensors—one upper-body, one lower-body—to capture full-body movement. By processing raw, normalized sensor data directly, this study aims to skip the heavy feature-extraction overhead and build a model that actually generalizes to new sessions for real-time smart home control. Ultimately, targeting high accuracy, balanced predictive power, fast computation, and clear model interpretability.

This article proposes the use of two wearable devices worn on the right ankle and wrist to perform sensor-based activity recognition. These devices are based on STM development boards. The data obtained are used to train classification models capable of recognizing nine human activities: standing in one location with minimal movement, writing with a pen and paper, climbing the stairs up and down, jogging, walking, using a vacuum cleaner, using a computer, and relaxing. The study investigates the performance of six common classification methods (Logistic Regression (LR), ANN, C5, Support Vector Machines (SVM), Random Trees (RT), and Random Forests (RF)), and the accuracy and flexibility of these methods were cross-examined. The suitable techniques will ultimately be used for live recognition and integration within smart home environments. The main objectives of using wearable sensors for smart home integration in this study are enabling accurate and continuous recognition of occupant activities in real time using unobtrusive body-worn sensors; improving robustness and reducing ambiguity between activities by capturing complementary upper- and lower-body movements through wrist and ankle sensor placement; and supporting adaptive smart home automation by translating recognized activities into context-aware control actions, such as lighting and climate adjustment. This study offers a novel methodology and integration with smart homes. The number and precision of recognizable activities in this study are superior to those of most similar and previous studies. The study investigates new classes of activities that are less widely explored, in addition to the integration with smart homes and intelligent buildings (through KNX), which will be explored. The main contributions of this article are summarized as follows:

- Complementary Dual-Body Kinematic Fusion: The article introduces a lightweight right-wrist and right-ankle sensor layout leveraging 9-DoF motion streams (accelerometer, gyroscope, and magnetometer sampled at 66.6 Hz) to resolve motion ambiguity across nine distinct daily activities.
- Raw Signal Processing Pipeline: The article shows that training classifiers directly on normalized, raw time-series windows (120 samples) eliminates the need for manual feature engineering without sacrificing model accuracy.
- Rigorous Cross-Dataset Generalization Analysis: We evaluate six machine learning models (SVM, LR, ANN, C&RT, RF, and C5.0) using strict cross-dataset validation on separate recording sessions, demonstrating why ANN and Random Forest handle real-world shifts far better than standard decision trees.
- Predictor Importance and Kinematic Mapping: The article maps feature importance across specific sensor channels and body placements for each activity class, revealing distinct kinematic markers—such as the heavy reliance on magnetometer data during stair movement.
- End-to-End Smart Home Deployment: The article deploys a functional, low-latency prototype that connects PMML model execution in a C# application to a KNX automation system (via Falcon.NET) and MQTT messaging, achieving sub 2s response times for context-aware control.

The next Section 2 reviews similar studies, primarily focused on comparing smartphones, vision, and body-worn solutions. Section 3 briefly describes the machine learning methods tested and used in this study. Section 4 describes the overall scheme of the proposed solution and the measurement methods and protocols. Section 5 presents the results of different machine learning methods and the detailed accuracy of different activity classes for the selected models. Section 6 and Section 7 are designated for a discussion of the results obtained and the guiding conclusions.

## 2 Related works

Reviewing recent studies has shown that there are major sport-related applications for human activity recognition to accurately estimate and monitor energy expenditure (Zhuang and Xue, 2019; Mekruksavanich and Jitpattanakul, 2022; Yin et al., 2024). This provides valuable information about the amount of energy an individual uses to maintain essential body functions due to physical activity. Information about user activities and behaviors can also be used as an indication of health status and the prevention of an unhealthy lifestyle. Correct recognition may also be used to create additional comfort in daily life.

Smartphones are widely integrated into our daily lives, and integrated sensors within these devices play an important role in making smartphones more functional. Accelerometer sensors are almost standard hardware for these devices. Data collected from the accelerometer can be processed to detect changes in movement. Another common sensor inside smartphones is the gyroscope, which measures orientation by gravity; it is typically used to detect the position and alignment of the device. Many features in the data collected from these sensors can be used to recognize the activities of users who carry this device (Saudi, 2022; Thakur et al., 2022; Voicu et al., 2019).

Vision-based approaches for video surveillance and fall detection systems typically use static cameras; however, more advanced applications require dynamic cameras, depth cameras, or multiview camera systems in combination with image processing methods and human activity recognition. Often hidden Markov models, conditional random fields, and neural networks are used to perform this task. Early research in this field was mainly focused on very basic human activities such as jumping, running, and waving hands (Zhang et al., 2017). However, more recent research has shifted to more advanced activities, including human-human or human-object interactions (Yang and Tian, 2017; Kamel et al., 2019; Gao et al., 2019; Putra et al., 2022).

The wearable devices are close enough to the user. Therefore, in addition to determining the user’s behavior, it also determines the user’s personalized behavior and cognitive data, which can identify the user’s intention (Qiu et al., 2022). Islam et al. (2022) reviewed and summarized human activity recognition methods based on neural network architecture, namely, convolutional neural networks. Table 1 provides a summary of the review of measurement approaches in the field of recognition of human activity. The comparison of numerical accuracy between these approaches is rather difficult as the number and type of recognizable activities greatly affect the numerical value.

**TABLE 1:** Summary of the review of three main categories of solutions for human activity recognition.

Approach	Accuracy	Advantages	Disadvantages
Smartphone-based Zhuang and Xue (2019); Mekruksavanich and Jitpattanakul (2022); Yin et al. (2024); Saudi (2022); Thakur et al. (2022); Voicu et al. (2019)	85%–95% (varies by features and models)	Widely available within most of the population. Good computation power within most devices. Deep learning ≤ 96%	Limited recognizable activities. Not always being carried by the user. Can be placed in a different location within the body
Vision-based Zhang et al. (2017); Yang and Tian (2017); Kamel et al. (2019); Gao et al. (2019); Putra et al. (2022)	70%–90%, (varies strongly by dataset and environment). Deep learning ≥ 90%	Larger recognizable activities and postures	Privacy concerns. Limited location and viewing angle. Lower accuracy. Need for large computational power. The requirement for additional devices. More sophisticated classification methods are required
Body-worn sensors-based Qiu et al. (2022); Islam et al. (2022); Banjarey et al. (2022); Khan et al. (2010); Zheng et al. (2018); Hysenllari et al. (2022)	90%–98%, smartwatch-based 92%–96%. Multi-sensor fusion ≥ 95%	Can be carried and worn the entire day. Small size. Can be used at multiple locations on the body. A larger number of recognizable activities. A larger number of devices can be used to recognize more activities. Availability using smartwatches	Limited computational power. Higher cost. The requirement of additional devices

Banjarey et al. (2022) introduced a technique that uses a one-dimensional convolutional neural network to recognize expected human behavior, obtaining 90.73% accuracy. Khan et al. (2010) expand their research to the recognition of human activity based on accelerometer sensors using a hierarchical scheme. The method recognized three states and 15 activities with an average accuracy of 97.9% using only a single triaxial accelerometer worn on the chest. Zheng et al. (2018) experimented with acceleration data-based human activity recognition from seven wearable devices, reaching an accuracy of 97.20% for eight daily activities. Hysenllari et al. (2022) presented a human activity recognition method using a convolutional neural network on acceleration and angular velocity data recorded from sensors at the ankle, thigh, hip, wrist, upper arm, and chest locations. The data obtained were used to recognize sitting, standing, lying, walking, jogging, and cycling. The accuracy of the network varied for different sensors in the range of 96.57% (ankle) to 99.28% (thigh).

Recent advances in sensor-based Human Activity Recognition (HAR) have emphasized extensive feature extraction and selection pipelines to improve classification performance. Viswalal and Poulose (2025) demonstrated that applying complex feature engineering alongside feature selection can significantly boost the accuracy of traditional classifiers like Linear SVC and Logistic Regression on benchmark datasets. However, relying on extensive offline feature extraction introduces notable computational overhead, increasing data processing latency and making real-time deployment challenging in constrained smart home environments. Furthermore, many existing studies optimize models using isolated benchmark datasets without testing cross-session generalization or evaluating model execution latency on real-world automation protocols. To avoid manual feature engineering, deep learning architectures have gained significant traction. Ronald et al. (2021) proposed iSPLInception, a lightweight Inception-ResNet architecture designed to extract rich spatial-temporal features directly from raw sensor streams while minimizing computational resource usage across public datasets like PAMAP2 and Opportunity. Although such deep architectures reduce manual preprocessing and achieve competitive accuracy, two critical research gaps remain unaddressed in the literature: the cross-session generalization gap and the end-to-end automation integration gap. This study directly bridges these gaps by proposing a raw-signal, dual-location (wrist and ankle) HAR framework, benchmarking 6 ML architectures under cross-session scoring, and deploying the optimal model into a functional, low-latency KNX/MQTT smart home control system.

Recent sensor signal classification increasingly balances accuracy, speed, and interpretability. Cacciola et al. (2026) combined fuzzy similarity learning with structured rules for acoustic diagnostics, achieving sub-millisecond (0.2 ms) inference. Although applied to structural monitoring rather than activity tracking, its focus on fast, interpretable, low-overhead inference directly aligns with our real-time smart home HAR framework.

In general, the review of state of art (Qiu et al., 2022; Islam et al., 2022; Banjarey et al., 2022; Khan et al., 2010; Zheng et al., 2018; Hysenllari et al., 2022) for the application of wearable devices showed higher accuracy and a greater number of recognizable activities. The general result of the state-of-the-art review is shown in Table 1. By combining six classification methods that include traditional machine learning and decision tree-based approaches, the system achieves over 99% accuracy with RF and ANN, demonstrating robust, real-time applicability for adaptive smart home automation, which establishes a foundation for expansion toward broader elderly monitoring, emergency detection, and personalized assistance.

## 3 Classification methods

Classification is the process of training machine learning algorithms to recognize patterns and assign class labels to data samples. There are numerous methods for performing classification, each suited to different types of problems. This section briefly describes six of the most common classification methods that are applied in this study. The first three methods are conventional machine learning algorithms (SVM, LR, and ANN), and the second half are decision tree-based methods (Classification and Regression Trees (C&RT trees), RF, and C5). LR or single decision trees typically require less computation and can provide near-instantaneous predictions, making them suitable for low-power devices. In contrast, more complex methods like RF and ANN, while achieving higher accuracy by capturing complex, nonlinear relationships in the sensor data, involve multiple tree evaluations or layered computations.

### 3.1 Support-vector machines

For classification purposes, SVM, also known as support vector networks, is a type of nonprobabilistic binary linear classifier that uses supervised learning models that are commonly applied to classify data into two different classes or for regression problems. To make the input data more separable compared to the original input space, non-linear kernel functions are often used to transform the input data into a high-dimensional feature space. The SVM model ibm (2022) used in this work can be described as follows: If $x_{i}$ is the i -th training sample, $y_{i}$ is the class label for the i -th training sample, and I is the number of samples such that the training vectors $x_{i}\in R^{i},\;i=1,\ldots ,I$ , in two classes, and a vector $y\in R^{i}$ such that $y_{i}\in \left\{-1,1\right\}$ , then the algorithm solves the following dual problem in $Minf\left(\alpha \right)=\frac{\alpha^{T}Q\alpha }{2}-e^{T}\alpha$ . Where $\alpha_{i}$ is the coefficient for the training sample and $0\leq \alpha_{i}\leq C,\;i=1,\ldots ,I$ such that $y^{T}\alpha =1$ , where $\alpha ={\left(\alpha_{1},\alpha_{2},\ldots ,\alpha_{I}\right)}^{T}$ and the kernel matrix is $Q\left(x_{i},x_{j}\right)=y_{i}y_{j}K\left(x_{i},x_{j}\right)$ then the decision function D is given by $D=\text{sgn }\left(\sum_{i=1}^{I}y_{i}\alpha_{i}K\left(x_{i},x\right)+b\right)$ , where b is a constant term.

### 3.2 Logistic regression

Regression is considered one of the classic supervised learning strategy algorithms in machine learning (Rencher and Christensen, 2012; Tolles and Meurer, 2016). Regression often comes in two types, linear and logistic, to solve regression problems and classification problems, respectively. LR applies the logistic function $f\left(x\right)=\frac{1}{1+e^{-k\left(x-x_{0}\right)}}$ ibm (2022), where values are mapped between 0 and 1. LR models only result in binary classification; when more than one class (or output) is required, multinomial LR is used. LR, by itself, is not typically regarded as a classifier, but it can function as a classifier by selecting a cutoff value between 0 and 1. Equation $y=\frac{1}{1+e^{-\left(\beta_{0}+\beta_{1}x_{1}+\beta_{2}x_{2}+\beta_{3}x_{3}+\cdots +\beta_{n}x_{n}\right)}}$ Represents the general formula for LR. Regression models can be trained using Stepwise, Enter, Forward Selection, and Backward Elimination, which dictate the way independent variables are included in the model. Regression models are usually tested using the Hosmer-Lemeshow test and the Omnibus test to assess the lack of fit and to determine whether the model outperforms the null model.

### 3.3 Artificial neural network

ANNs are widely adopted classification methods due to their ease of use, flexibility, and high accuracy Jamali et al. (2025). The models consist of a learning process and the strengths of connections between neurons. The multilayer perceptron is among the most widely used types of feedforward ANNs that utilize backpropagation for training Chauhan et al. (2024). With nonlinear activation functions and multiple hidden layers, it can distinguish non-linearly separable data. This study uses a MLP ANN that includes an input layer, a single hidden layer, and output layers, so that its mathematical model ibm (2022) can be described as follows: The input layer consists of $j_{0}=p$ units, $a_{0:j},\ldots ,a_{0:j_{0}}$ , with $a_{0:j}=x_{j}$ , j is the number of neurons in the input layer and X is the input. Each ${\text{i}}^{\text{th}}$ hidden layer consists of $j_{i}$ units, $a_{i:1},\ldots ,a_{i:j_{i}}$ , with $a_{1:k}=\gamma_{i}\left(C_{i:k}\right)$ and $C_{i:k}=\sum_{j=0}^{j_{i-1}}\omega_{i:j1}k^{a_{i-1:j}}$ , where $a_{i-1:0}=1$ , $\gamma_{i}$ denotes the activation function for layer i , and $\omega_{i:j1}$ refers to the weight connecting to the next layer i−1 . At this layer, for the hidden layers, the hyperbolic tangent activation function $\left(\gamma \left(C\right)=\tanh\left(c\right)=\frac{e^{c}-e^{-c}}{e^{c}+e^{-c}}\right)$ is used. The output layer consists of $j_{I}=R$ units, $a_{I:1},\ldots ,a_{I:J_{I}}$ , where $a_{I:k}=\gamma_{I}\left(C_{I:k}\right)$ and $C_{I:k}=\sum_{J=0}^{J_{1}}\omega_{I:j},k^{a_{i-1:j}}$ , where $a_{i-1:0}=1$ . The activation function used in the output layer is SoftMax. $\left(\gamma \left(C_{k}\right)=\frac{e^{c_{k}}}{\sum_{j\in \Gamma_{h}}e^{c_{j}}}\right)$ .

### 3.4 Random tree - classification or regression trees (C&RT)

Decision trees are a popular method for machine learning tasks. Decision trees are often used as a predictive model Parhi and Patro (2024). C&RT is a variant of decision trees that offers multiple possibilities for the output variable. It is a sophisticated and modern tree-based supervised learning classification that relies on majority voting, where an ensemble model is created by combining multiple decision trees. The C&R (classification and regression) trees are binary models with inner splitting conditions Coulston et al. (2016). The RT models are less prone to overfitting due to the use of bagging and field sampling. It is commonly applied in situations that involve large datasets and numerous fields. The algorithm uses the available input variables to find the best split as a threshold value of an attribute determined by the best homogeneity for the sub-nodes, using the Gini index criterion. The process continues until the last pure subset is found in the tree or the maximum limit of leaves is attained. The equation of the Gini index is given by $GI=1-\sum_{i=0}^{c}P_{i}^{2}$ ibm (2022), where C is the total number of classes, while P is the probability of class i .

### 3.5 Random forest

RF models are based on a large number of individual decision trees that work together as an ensemble in which each of them spits a class prediction, and then the class that receives the most votes becomes the model’s prediction. Essentially, it is based on the idea of crowd wisdom: numerous uncorrelated models that outperform any of the individual trees. Very deep trees are highly capable of learning irregular patterns, but sometimes tend to overfit the training sets with low bias but large variance. It averages numerous deep decision trees, resulting in lower variance and ultimately higher accuracy of the final model. In classification trees, the majority vote is used instead. The uncertainty of the prediction can be estimated using the standard deviation for each regression tree on x’ $\left(\sigma =\sqrt{\frac{\sum_{b=1}^{B}{\left(f_{b}\left(x^{\prime }\right)-\hat{f}\right)}^{2}}{B-1}}\right)$ ibm (2022), Coulston et al. (2016).

### 3.6 C5

The C5.0 algorithm is one of the most well-known types of decision tree-based classification. It generally performs nearly as well as neural networks and support vector machines, but is much easier to understand and deploy Rahi et al. (2024). The C5.0 algorithm performs many of the decision-making automatically with reasonable defaults. It first creates a large overfitting tree, and then it removes nodes and branches that have an insignificant effect on the classification errors. The Entropy concept is used to measure purity, where 0 indicates a completely homogeneous sample, and one indicates the maximum disorder. The trees can continue to grow indefinitely until each example is perfectly classified. However, overly-grown trees tend to overfit the training data. The records are assigned a score based on the class and confidence of the rule that is triggered for each record. The confidence of the rule is determined by the number of correct predictions in the leaf+1 divided by the total number of records+2 ibm (2022).

## 4 Measurement methods and protocols

The measurement system used in this study is based on two wearable devices worn on the right wrist and the right ankle to monitor sensor-based activity. The right wrist and right ankle were selected as sensor locations to capture complementary upper and lower body movements while maintaining simplicity and wearability. The wrist sensor is an effective location for recognizing arm-dominated activities and hand motion, and the ankle sensor captures posture and gait movements. Using these locations significantly reduces ambiguity between activities that may appear similar when monitored from a single body location. In addition, the wrist and ankle are easy to wear and consistent with common wearable accessories. These devices are based on STMicroelectronics development boards measuring the data from the gyroscope, accelerometer, and magnetometer using sensors onboard, which capture linear acceleration, angular velocity, and magnetic field orientation along three orthogonal axes, resulting in nine degrees of freedom (9-DoF) motion data:

- Accelerometer: ± 16 g measurement range, 16-bit resolution, 66.6 Hz sampling rate
- Gyroscope: ± 2000°/s, 16-bit resolution, 66.6 Hz sampling rate
- Magnetometer: ± 4800 μT, 16-bit resolution, 66.6 Hz sampling rate

Devices were worn on the right wrist and right ankle, securely fastened to minimize sensor movement relative to the body. The orientation of the sensors was standardized across participants to ensure reproducibility.

The devices send the measured data to the host computer in real time using WIFI technology, where the host computer performs data buffering and data processing (Figure 1). KNX Association offers libraries and support for the. NET Framework, and C# was used for development as described in Figure 1. Multiple libraries were used to establish a real-time connection. Machine learning was performed in IBM SPSS Modeler 18.5, and the models were exported as PMML-based files. The C# application imports these models, extracts the normalization and weight matrices to perform classification, and forwards the results. Figure 1 shows the simplified interaction of different components of the proposed solution. The rule-based decision-making system forwards commands to the KNX bus depending on the occupant activity to control lighting (lights and blinds) with ambient temperature according to preferences set by the occupants previously.

**FIGURE 1:**
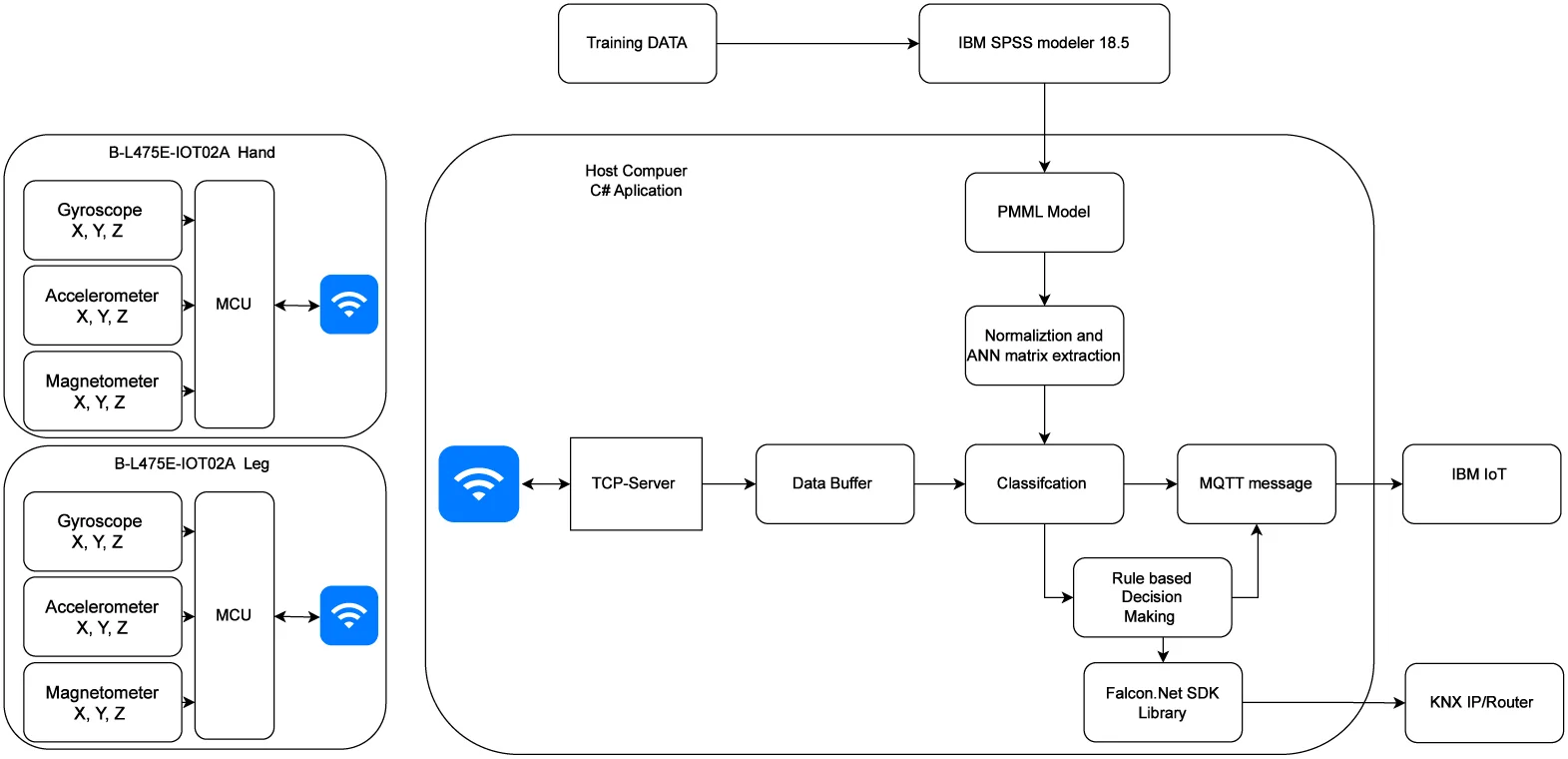
Simplified Block Diagram of the solution.

### 4.1 Data collection

The data obtained were used to train classification models that are capable of recognizing nine human activities (Table 2). These activities present a range of samples for activities with different intensities and movement patterns, making them suitable for testing real-time recognition systems and applications such as health monitoring, fall detection, and adaptive smart home automation. The type of included human activities directly impacts classification performance; different activities produce motion patterns of varying complexity and similarity. Activities with clear and consistent movements, such as jogging or relaxing, are easier for algorithms to classify, while overlapping motions, such as walking versus vacuum cleaning or writing versus using a computer, are more complex.

**TABLE 2:** Description of activity categories.

Activity class	Description
Class 1	Standing in one location with minimal movement
Class 2	Writing using a pen and paper
Class 3	Climbing the stairs case down
Class 4	Climbing the stairs case up
Class 5	Jogging
Class 6	Walking
Class 7	Cleaning floors using vacuum cleaner
Class 8	Using a computer for web surfing, light activity
Class 9	Relaxing-lying down with the minimal movements

Two independent datasets were collected, both of which included the same nine predefined activity classes. Each dataset contained three male test subjects with different ages (25–32), heights (180–190 cm), weights (88–125 kg), and fitness levels. Participants performed nine predefined activities (Table 2) in a controlled indoor environment for a duration of 5–10 min, at a sampling frequency of 66.6 Hz, resulting in two datasets recorded independently. Test subjects were given written consent to participate in the experiment, and the labeling was performed in real-time by experiment conductors to reduce ambiguity at activity transitions and improve data quality for reliable training and evaluation of machine learning models. The size and quality of the collected dataset, as well as accurate labeling, would strongly impact the performance of all classification methods, especially data-hungry methods like ANN, which can learn complex patterns and generalize better to unseen data, thereby reducing misclassifications and increasing robustness. Informed consent was obtained from all subjects who participated in this research. The collected data can be obtained from IEEE DataPort; see Gorjnai (https://doi.org/10.21227/08XK-R309, 2024). Figure 2 shows one example of measured signals from the dataset (the selected example relates to walking).

**FIGURE 2:**
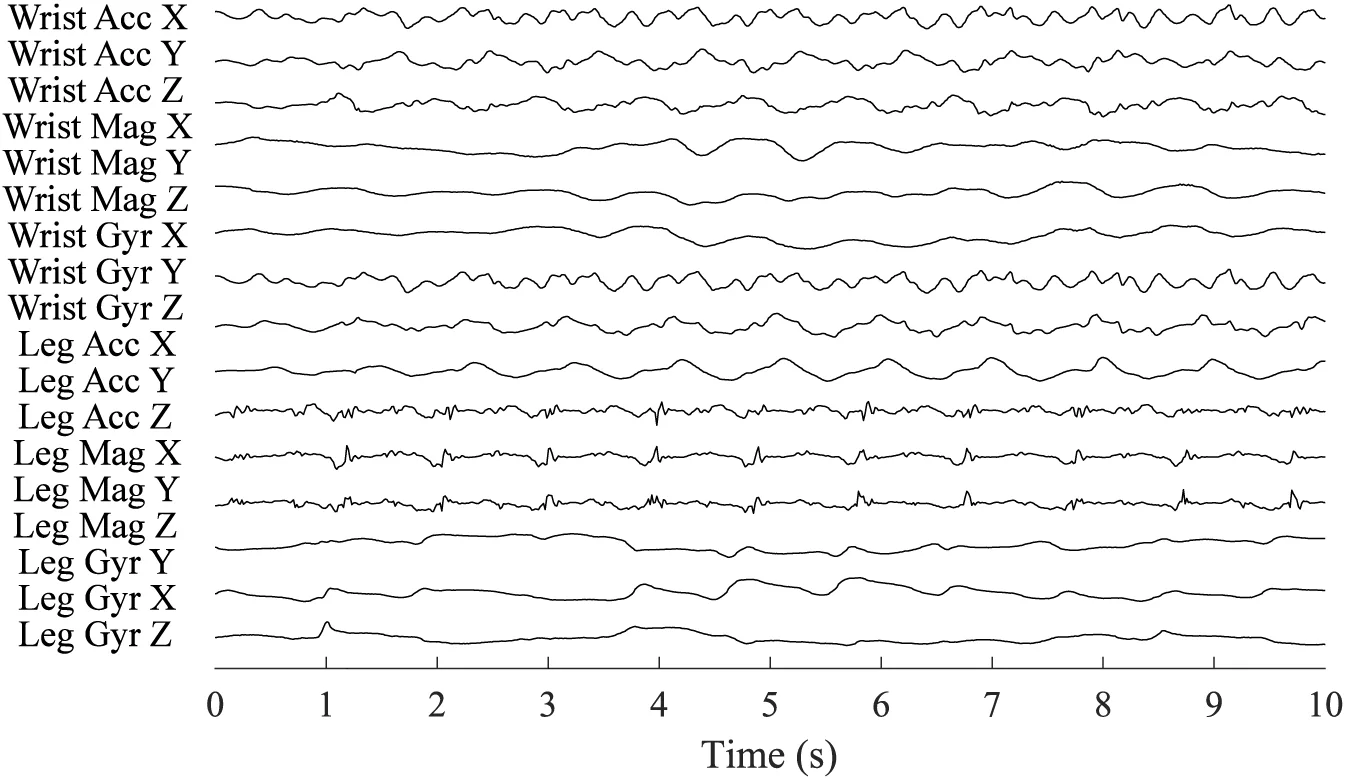
Measured signals example (walking).

### 4.2 Pre-processing

In this study, raw sensor signals from the wrist and ankle were used directly for model training without explicit feature extraction. Using raw data allows the models, especially ANN and RF, to automatically learn relevant patterns from sensor signals without hand-crafted features. Each 120-sample segment ( ≈2 seconds) of accelerometer, gyroscope, and magnetometer data was treated as the input vector for the machine learning models. Before training, each sensor channel was normalized to zero mean and unit variance. The collected datasets were divided into three subsets: training (40%), validation (30%), and testing (30%). For cross-dataset scoring, models trained on one dataset were evaluated on an entirely independent dataset recorded on a separate day. Given raw sensor channels $x_{i}\left(t\right)$ across 9-DoF streams (accelerometer, gyroscope, and magnetometer), each channel is standardized via Z-score normalization prior to window segmentation to eliminate sensor bias and scale disparities, see Equation 1.

$${\hat{x}}_{i}\left(t\right)=\frac{x_{i}\left(t\right)-\mu_{i}}{\sigma_{i}}$$ (1)

where $\mu_{i}$ and $\sigma_{i}$ denote the mean and standard deviation of channel i calculated across the training set.

Continuous multi-channel normalized signals $X\left(t\right)\in R^{d}$ (where d=18 channels across wrist and ankle) are segmented using a sliding window of length N=120 samples ( 1.8 seconds at 66.6 Hz ) with an overlap factor o∈[0,1) , see Equation 2:

$$W_{k}=\left[\begin{array}{cccc} {\hat{x}}_{1}\left(t_{k}\right) & {\hat{x}}_{2}\left(t_{k}\right) & \cdots & {\hat{x}}_{d}\left(t_{k}\right)\\ {\hat{x}}_{1}\left(t_{k}+1\right) & {\hat{x}}_{2}\left(t_{k}+1\right) & \cdots & {\hat{x}}_{d}\left(t_{k}+1\right)\\ \vdots & \vdots & \ddots & \vdots \\ {\hat{x}}_{1}\left(t_{k}+N-1\right) & {\hat{x}}_{2}\left(t_{k}+N-1\right) & \cdots & {\hat{x}}_{d}\left(t_{k}+N-1\right) \end{array}\right]$$ (2)

### 4.3 Classification

The classification was performed using six different classification methods with IBM SPSS Modeler software. These six methods differ in their approach, computational complexity, and suitability for sensor-based human activity recognition. Support Vector Machine handles high-dimensional and nonlinear data effectively, but can be sensitive to noise and less interpretable. 1t is Suitable for small to medium datasets with clear class separation. Logistic Regression is Simple and interpretable; it performs well for linearly separable problems, but struggles with complex, nonlinear patterns in sensor data. Artificial Neural Networks can learn complex, nonlinear relationships, making them highly accurate, especially for multimodal sensor data. Training can be computationally intensive, but it scales well with data. Random Tree is a single decision tree; simple and interpretable, robust to noisy data, but prone to overfitting and less accurate on complex datasets. A Random Forest Ensemble of decision trees reduces overfitting, improves generalization, and is robust to noise, providing feature importance measures. More computationally intensive than RT. C5.0 is an advanced decision tree algorithm that performs pruning to avoid overfitting. It is relatively easy to interpret and often performs close to an ANN in accuracy. However, it may struggle with subtle activity differences compared to ensemble methods. Using multiple methods allows comparison of accuracy, generalization, and robustness, helping to identify the most suitable algorithm for real-time smart home applications. For the Artificial Neural Network (ANN/MLP) classifier, the input vector $w_{k}=\text{vec}\left(W_{k}\right)\in R^{N\times d}$ is mapped to C=9 target activity classes via non-linear transformations across hidden layers, see Equation 3.

$$h^{\left(l\right)}=f\left(W^{\left(l\right)}h^{\left(l-1\right)}+b^{\left(l\right)}\right)$$ (3)

where f(⋅) represents the activation function (e.g., ReLU), $W^{\left(l\right)}$ and $b^{\left(l\right)}$ are layer weights and biases, and output probabilities are obtained using the Softmax function, see Equation 4.

$$P\left(y=c|w_{k}\right)=\frac{e^{z_{c}}}{\sum_{j=1}^{C}e^{z_{j}}}$$ (4)

The network parameters are optimized by minimizing the categorical cross-entropy loss function L , see Equation 5.

$$L=-\overset{C}{\underset{c=1}{\sum }}y_{c}\log P\left(y=c|w_{k}\right)+\lambda \underset{l}{\sum }\|W^{\left(l\right)}{\|}_{2}^{2}$$ (5)

where $y_{c}$ is the one-hot encoded ground truth label and λ is the $L_{2}$ regularization hyperparameter.

### 4.4 Real-time majority voting post-processing

To eliminate transient classification spikes during real-time KNX inference, predictions over a sliding evaluation buffer of M consecutive windows are smoothed using majority voting, see Equation 6.

$${\hat{Y}}_{\text{filtered}}=\arg\underset{c\in \left\{1,\ldots ,C\right\}}{\max}\overset{M}{\underset{m=1}{\sum }}I\left({\hat{y}}_{k-m+1}=c\right)$$ (6)

where I(⋅) is the indicator function returning one if the predictthirdss $\hat{y}$ equals c , and 0 otherwise.

### 4.5 Pseudo-code logic for end-to-end real-time pipeline

Algorithm 1 details the step-by-step logic of the proposed HAR system, tracing data execution from raw dual-sensor ingestion to real-time KNX actuation.

Algorithm 1. End-to-End Raw-Signal HAR Processing and Real-Time KNX Actuation.

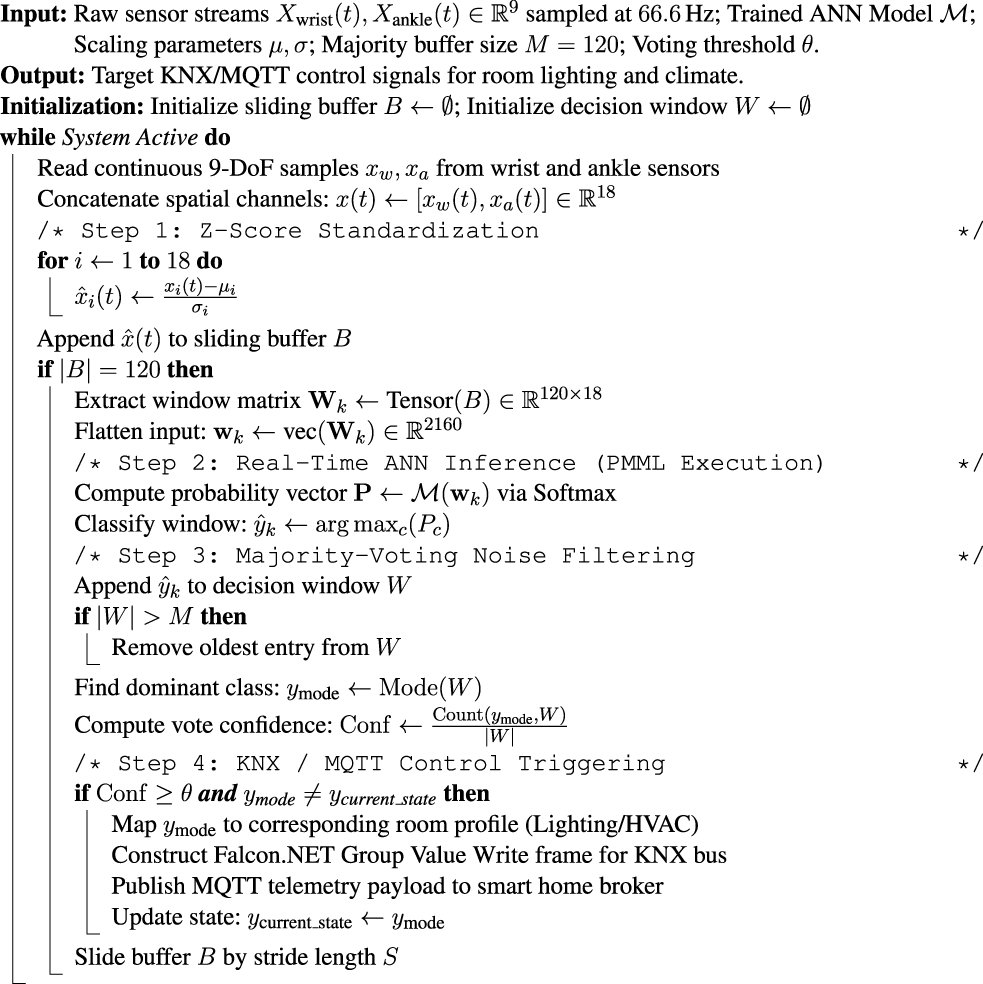

## 5 Implementation and results

New datasets were recorded using multiple test subjects on different dates, forming two datasets (Gorjnai, 2024). Figure 3 shows the placement of the measurement devices and the coordination of the sensors.

**FIGURE 3:**
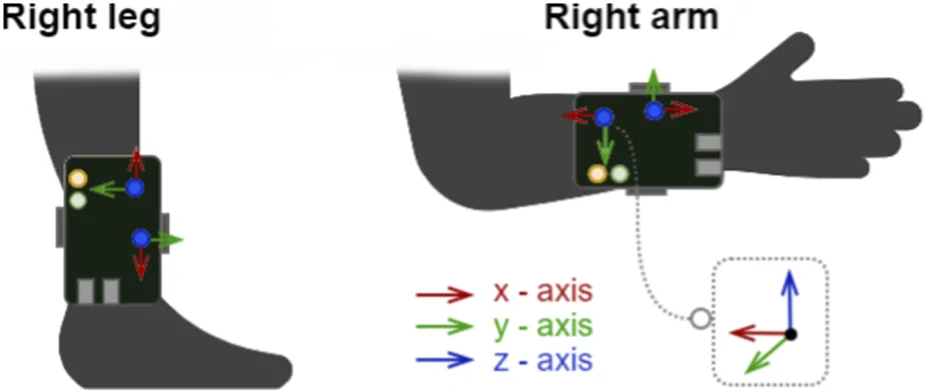
The location and orientation of the sensors.

Each dataset was individually divided into three segments: training, testing, and validation. This process is standard practice for testing models. This study takes the analysis a step further by using scoring accuracy, where models are trained and examined with entirely different datasets. The scoring accuracy is expected to be lower, but it is a good indication of how the models will perform in a real-time application. This section provides the accuracy of each method in terms of testing and scoring.

The analysis was carried out with IBM SPSS Modeler, which uses previously explained mathematical equations to develop its classification models. The initial phase involved normalization of datasets and training and assessment through testing. The datasets were divided into three subsets of training, testing, and validation (40%-30%-30%, respectively), and analyzed using different methods. For more accurate comparability between binary and multi-class model learning methods, the average accuracy across classes was selected as the main evaluation metric. Table 3 shows the average accuracy of each classification method tested. The second column shows the average testing accuracy across all activities, and the third column shows the average scoring accuracy across all classes where the trained models were evaluated with entirely different datasets. The SVM showed the highest levels of average testing accuracy (99.73%) and showed a very high average scoring accuracy of 91.26%, which is more than an acceptable result. Meanwhile, LR showed the lowest testing accuracy with an average testing accuracy of 94.44% for correct predictions but scored above 90%, which is still an acceptable outcome, too. Among tree-based methods, the RT method resulted in the lowest testing average accuracy (97.79%) and the highest scoring (88.27%). The C5 method showed accurate testing (average 99.53%) but did not score well (88.13%), indicating that the trained model likely overfits the training data. On the other hand, the RF method was tested on average at 99.70% while having a good scoring accuracy of 92.18%. Therefore, it is the most suitable method among tree-based methods. Lastly, ANN were tested to average above 99% and scored the best (93.48% correct predictions) among all methods. Given the small size of neural networks, this accuracy can increase significantly with larger neuron counts. A closer look at the scoring results of this method revealed that it performs consistently well across all activity classes. In general, RF and ANN can be assumed to be the best two methods for recognizing human activity based on sensors.

**TABLE 3:** Average testing and scoring accuracy among the classification methods.

Activity class	SVM			LR			ANN		
	Testing (D1)	Testing (D2)	D1XD2	Testing (D1)	Testing (D2)	D1XD2	Testing (D1)	Testing (D2)	D1XD2
Class 1	100.00%	100.00%	100.00%	99.95%	100.00%	99.99%	99.98%	100.00%	99.99%
Class 2	100.00%	99.97%	80.87%	99.04%	95.38%	84.59%	99.96%	99.94%	95.11%
Class 3	99.68%	99.31%	90.34%	97.42%	97.23%	96.84%	98.75%	98.23%	95.95%
Class 4	99.72%	99.50%	98.04%	97.11%	97.82%	97.15%	98.60%	98.92%	97.43%
Class 5	99.77%	99.76%	97.07%	92.30%	95.66%	93.81%	98.60%	98.81%	96.86%
Class 6	99.89%	99.86%	89.15%	89.67%	88.70%	86.81%	99.79%	99.38%	90.78%
Class 7	99.60%	99.64%	80.06%	92.47%	93.75%	82.60%	98.57%	98.47%	85.54%
Class 8	99.25%	99.08%	94.39%	86.78%	85.90%	88.03%	96.90%	97.83%	90.66%
Class 9	100.00%	99.94%	87.69%	94.37%	96.95%	82.79%	99.93%	99.92%	90.98%
Average	99.77%	99.67%	90.85%	94.35%	94.60%	90.29%	99.01%	99.06%	93.48%

Activity class	RT			RF			C5		
	Testing (D1)	Testing (D2)	D1XD2	Testing (D1)	Testing (D2)	D1XD2	Testing (D1)	Testing (D2)	D1XD2
Class 1	100.00%	100.00%	95.50%	100.00%	100.00%	99.99%	99.83%	99.75%	89.37%
Class 2	99.95%	99.85%	76.77%	99.99%	99.99%	79.64%	99.96%	99.96%	77.43%
Class 3	96.99%	93.80%	92.06%	99.55%	99.25%	97.72%	99.36%	99.10%	95.12%
Class 4	96.41%	97.61%	92.72%	99.59%	99.55%	98.13%	99.61%	99.51%	86.58%
Class 5	95.38%	97.08%	92.61%	99.51%	99.63%	97.78%	99.14%	99.26%	94.84%
Class 6	99.18%	99.40%	94.63%	99.93%	99.85%	88.98%	98.81%	98.63%	94.70%
Class 7	97.71%	96.92%	79.08%	99.67%	99.64%	86.17%	99.36%	99.26%	84.57%
Class 8	95.19%	95.12%	85.22%	99.36%	99.20%	95.85%	99.98%	99.96%	79.54%
Class 9	99.98%	99.93%	85.82%	99.98%	100.00%	85.34%	99.99%	100.00%	99.99%
Average	97.87%	97.75%	88.27%	99.73%	99.68%	92.18%	99.56%	99.49%	89.13%

Figure 4 graphically presents the results of Table 3 in the form of boxplots. The boxplots show that the methods generally exhibit high accuracy when tested on individual datasets (D1, D2), but most classifiers show a significant drop in performance when scoring data from D2 using a model trained on D1. The SVM and RF methods achieve the highest and most stable performance in all test scenarios. However, when scoring alone is considered, we designate the RF and ANN methods as the most effective.

**FIGURE 4:**
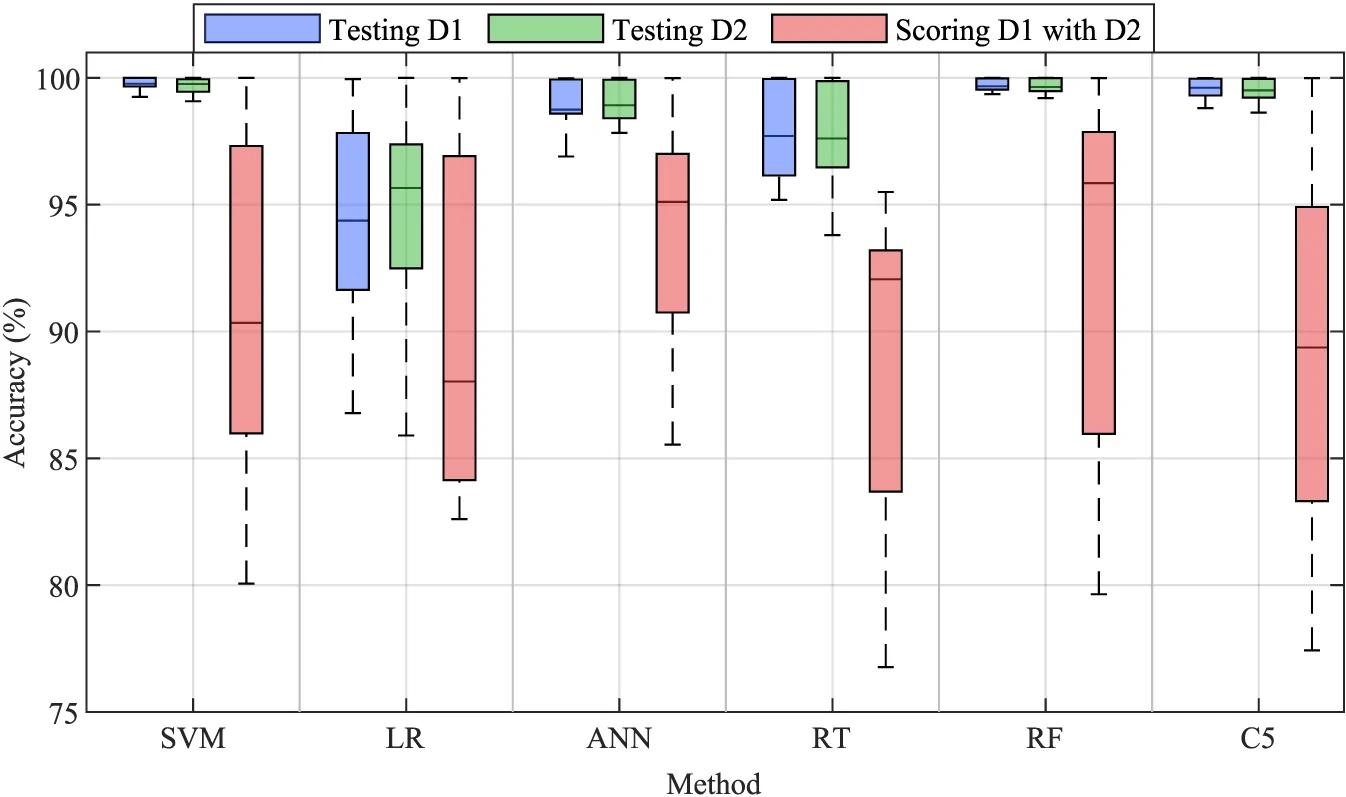
Visual demonstration of testing and scoring accuracy of different classification methods.

ANN was selected as the most suitable method, and an MLP model that employs just one hidden layer was used for method selection; the hidden layer neuron count was set to 8. The smaller neuron count resulted in a relatively high training speed while presenting very accurate results. Only two stopping criteria were used for the maximum training time was reached or when accuracy cannot be further improved. Given the small size of the models, the training of all models stopped when the accuracy could not be further improved. Furthermore, the number of components for posting or bagging was set to 10. The results of the tests and the scores are shown in Table 4. The average testing accuracy of datasets D1 and D2 were 99.01% and 99.05%, respectively, which was slightly lower than SVM but noticeably higher than LR. Similarly to other methods, class 1 was the highest and class 8 the lowest testing class. The results are more consistent across different activities. Class 7 vacuum cleaning is a bit more challenging to recognize due to inconsistency within the activity and the movements of the test subjects. Considering the scoring accuracy of each activity class, this method can be recognized as the most suitable for sensor-based human activity classification applications. Furthermore, the accuracy levels can be further increased by using a larger neural network model with a higher neuron count. The scoring average of 93.8% surpasses the state of art, especially given that this approach classifies a large number of complex activities (9 classes).

**TABLE 4:** Training, validation, testing, and scoring accuracy of ANN Model.

Activity class	Dataset D1			Dataset D2			D1XD2
	Training	Testing	Validation	Training	Testing	Validation	
Class 1	99.99%	99.98%	99.98%	100%	100%	100%	99.99%
Class 2	99.97%	99.96%	99.96%	99.95%	99.94%	99.93%	95.11%
Class 3	98.73%	98.75%	98.69%	98.26%	98.23%	98.18%	95.95%
Class 4	98.63%	98.60%	98.60%	98.92%	98.92%	98.92%	97.43%
Class 5	98.63%	98.60%	98.60%	98.82%	98.81%	98.83%	96.86%
Class 6	99.79%	99.79%	99.73%	99.38%	99.38%	99.37%	90.78%
Class 7	98.63%	98.57%	98.63%	98.54%	98.47%	98.48%	85.54%
Class 8	97.03%	96.90%	96.96%	97.87%	97.83%	97.82%	90.66%
Class 9	99.94%	99.93%	99.92%	99.94%	99.92%	99.92%	90.98%
Average	99.04%	99.01%	99.01%	99.08%	99.06%	99.05%	93.48%

Figure 5 shows the average classification accuracy with the increase in the hidden layer size. The plot clearly shows an increase in accuracy with the increase in neuron count up to 128 neurons, and it declines rapidly after 256 neurons as either overfitting occurs or the training time limitation prevents fully trained models.

**FIGURE 5:**
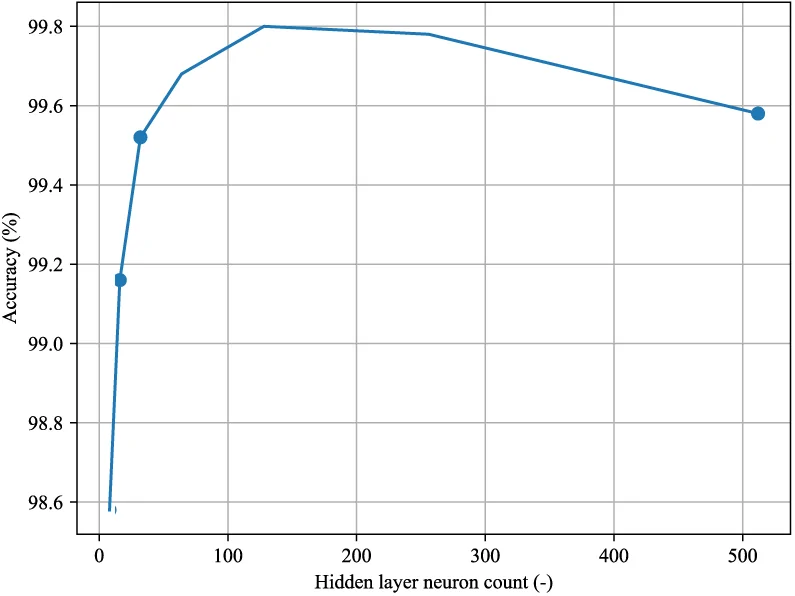
The effect of hidden layer neuron count on average testing accuracy.

The above results show exceptionally accurate recognition and highlight the strong potential of the proposed method. To develop an estimation of the real-time accuracy of the models, they were scored against alternative datasets. As mentioned earlier, significant differences in accuracy levels are expected compared to the testing results. A more significant discrepancy indicates a greater need for a larger training dataset.

In the next stage, the importance of predictors was investigated. Climbing the stairs down and up both activities show similar relations to the sensor data. In both models, the leg magnetometer across the Y-axis is the most important predictor, as it can sense the height difference in rotation. In general, the first three important predictors are based on magnetometer data for both activities. Using a personal computer and writing with a pen both show a high correlation with hand movements, as the most important predictors are based on wrist accelerometer data. However, the direction is different. Inactivity of the legs during these activities is also an important factor in the correct prediction. The inactivity of the test subject during this activity class of relaxing with minimal movements is the most important feature of the data. The most important predictors for this activity are based on accelerometers and a magnetometer located on the ankle of the test subject. A similar characteristic could be observed in the standing activity class. However, wrist sensors play a slightly more significant role as they help the model distinguish between standing and relaxing activity classes. Running activity showed a good balance between wrist and ankle sensors, as running involves hand and leg movements. Similarly, walking also involves hand and leg movements. However, the importance of predictors is less balanced in comparison to running activity. This is due to the different nature of body movement while walking. The vacuum cleaning activity involves very complex movements of the body, and it is very challenging for most models to provide a good classification. From the predictor importance chart (Figure 6), horizontal movements of the arm are the highest signature of this activity, but sensors on the ankle also play a role in the recognition of this activity.

**FIGURE 6:**
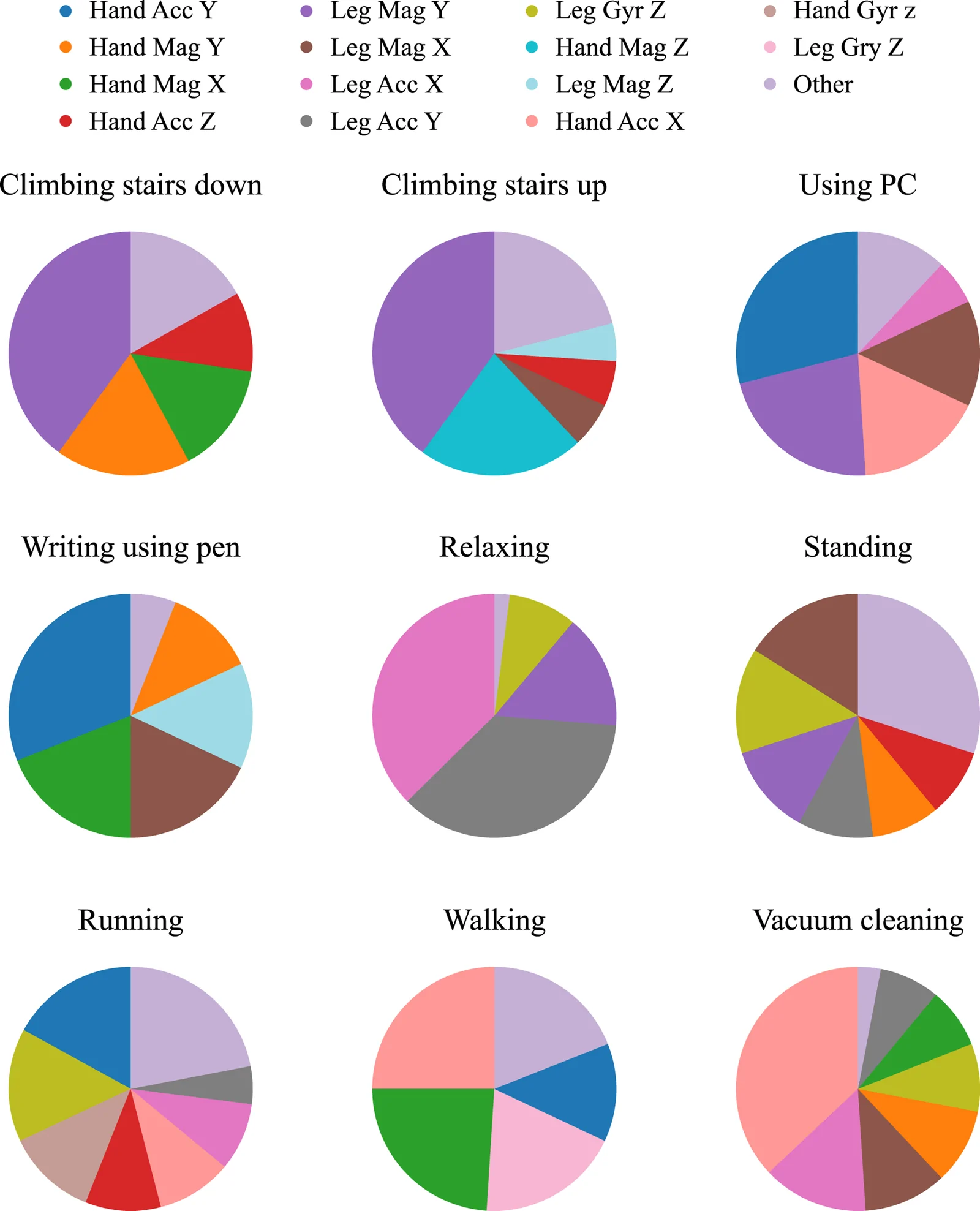
Predictors importance for different activity classes.

The models were imported into the developed software as described in Figure 6. To improve recognition accuracy, outliers were detected by using the majority voting technique or a median filter. The data were divided into segments with a size of 120 records (which is approximately 2 s in real-time recognition), and the activity type with the most occurrences was selected as the segment’s outcome. This method eliminates prediction noise (minor false detections), but it introduces additional false recognition at the moment of activity change; given the non-sensitive nature of the data, a slight delay in recognition is negligible. In other words, with a buffer size of 120 samples and a sampling frequency of 66.6 Hz, the recognition delay will be less than 2 s in real-time applications with the advantage of eliminating the impact of some minor deviation of activity such as a single hand swing during the walking activity. Real-time testing of the solution showed results similar to scoring.

To assess how well the proposed framework handles real-time deployment, the latency, memory usage, CPU load, node power consumption, and overall system stability on a C# host machine (Intel Core i5-1135G7 @ 2.40 GHz, 16 GB RAM) linked to a physical KNX IP Interface via Falcon.NET was measured. As shown in Table 5, processing and communication add 15.0 ms of overhead to the required 1.8-s sensor sampling window. These results confirm that the PMML execution engine easily manages real-time KNX smart home control with minimal impact on system resources.

**TABLE 5:** Quantitative real-time system performance and resource footprint.

Performance category	Metric/Indicator	Measured value/Detail
Processing latency	Sensor window assembly (N=120)	1,800.0 ms (physical sampling duration)
	Z-score normalization and stride prep	0.4 ms
	C# PMML neural network inference	1.8 ms
	Majority-voting filter (M=120)	0.1 ms
	Falcon.NET/KNX bus transmission	12.7 ms
	Total end-to-end system latency	1,815.0 ms ( <2.0 s target)
Resource consumption (Execution host)	C# host process memory footprint	41.8 MB RAM
	Host CPU utilization	0.8%–1.2% (single core peak)
	PMML file storage footprint	142 KB
Wearable node power	Node operating voltage/Current	3.7 V/28.4 mA (active 66.6 Hz streaming)
	Estimated battery endurance	≈14.0 hours (400 mAh LiPo cell)
Operational stability	Continuous test trial duration	12.0 h continuous run
	Memory leakage/Thread exceptions	0 events detected
	KNX bus telegram delivery rate	100% (0 packet drops)

## 6 Discussion

In this work, two wearable devices were used to capture and record the movements of test subjects. Placing sensors on the wrist and ankle allows the system to capture both upper-body movements (wrist) and lower-body movements (ankle), which helps reduce confusion between activities that may appear similar when using a single sensor. This dual placement improves classification performance by providing complementary information, making activity recognition more robust to variations in movement patterns across different individuals. Furthermore, these sensor positions are easy to wear and correspond to common accessory locations, ensuring comfort and practicality for real-world use. The data obtained were classified using three statistical machine learning methods (SVM, LR, and ANN) and three tree-based decision-making methods (RT, RF, and C5). Decision tree-based methods, RT, C5, and RF are generally robust to noisy or missing data, while RF is less prone to overfitting and offers better generalization. SVM can handle high-dimensional or nonlinear data while being sensitive to noise and missing data. LR is simple, but it struggles with complex patterns. ANN can learn complicated relationships in the data, picking up subtle patterns that simpler models might miss, especially when using combined signals from the ankle and wrist. Due to these varying strengths and weaknesses, the comparison of multiple algorithms is a valuable and important segment of the work.

These methods classified the records into nine classes: standing in one location with minimal movement, writing using a pen and paper, climbing the stairs case down, climbing the stairs case up, jogging, walking, cleaning floors using the vacuum cleaner, using a computer for web surfing, and relaxing-lying down with minimal movements. These activities were chosen to represent a range of common daily behaviors involving both upper- and lower-body movements to include a base-line, mobility and upper body or household actions. The selection was limited to tasks that can be reliably captured using wearable sensors on the ankle and wrist.

Model training, evaluation, and hyperparameter optimization were executed within IBM SPSS Modeler. While open-source Python ecosystems (e.g., PyTorch, TensorFlow) offer wider library support for custom sequential deep learning models (such as LSTMs or Transformers), IBM SPSS Modeler was intentionally selected due to its standardized Predictive Model Markup Language (PMML) export pipeline. Exporting the trained Multi-Layer Perceptron (ANN) and Random Forest models as structured PMML XML files enabled direct, native execution within a custom C# host application. This design eliminated the need to embed external Python runtime environments or inter-process communication wrappers on the building automation server, ensuring a low-latency ( <2 s) and deterministic execution pipeline via the Falcon.NET KNX driver framework.

A close look at the analysis showed that relaxing lying down with minimal movements) is the most accurate class among all methods, and cleaning floors using a vacuum cleaner is the hardest activity to classify, as it scored lowest among all methods. A common trend within the scoring results showed that vacuum cleaning is often confused with walking, as it shares similar movements. Also, it can be easily noted that the decision tree-based methods did not handle class 8 (using a computer activity) scoring as well as SVM, LR, and ANN; sometimes, it was recognized as writing using pen and paper due to the similar posture of the test subjects. Overall, as it was expected, movements with distinct and consistent patterns, such as jogging or relaxing, present easier-to-classify data, while activities with overlapping or similar motions such as walking versus vacuum cleaning or writing versus using a computer are more prone to ambiguity and misclassifications. Testing all models yielded an average accuracy of 98.37% and a scoring accuracy of 90.70%; the small differences between these two values indicate that the data size used in this work is sufficient.

Support-vector machines showed the highest accuracy levels (99.73%) and showed a very high average scoring accuracy of 91.26%, which is more than an acceptable result. Meanwhile, LR showed the lowest testing accuracy with an average of 94.44% for correct predictions but scored above 90%, which is an acceptable outcome too. The RT method resulted in the lowest testing (97.79%) and scoring (88.27%) accuracy among tree-based methods. The C5 method showed accurate testing (average 99.53%) but did not score well (88.13%); this indicated that the trained model is likely over-fitted to the training data. On the other hand, the RF method tested 99.70% while holding a good scoring accuracy of 92.18%. Therefore, it’s the most suitable method among tree-based methods. Lastly, ANNs were tested to average above 99% and scored best (93.48% correct predictions) among all methods. Given the small size of neural networks, this accuracy can increase significantly with larger neuron counts. A closer look at the scoring results of this method revealed that it performs consistently well across all activity classes. In general, RF and ANN can be assumed to be the best two methods for recognizing human activity based on sensors. Real-time testing of the solution showed results similar to scoring and successful integration with smart homes. The accuracy advantage of RF and ANN can be explained by their superiority in handling variability between subjects and distinguishing overlapping activities.

Overall, decision tree-based methods achieved testing accuracy comparable to traditional machine learning approaches. However, when evaluated on independent datasets, simpler tree-based models showed a noticeable drop in performance, suggesting sensitivity to dataset-specific patterns. In contrast, Random Forest and nonlinear models such as ANN generalized more reliably across datasets, as they are better able to handle variability between subjects and capture complex, overlapping motion patterns directly from the raw sensor data. These properties make RF and ANN more effective and better suited for wearable sensor-based human activity recognition in real-world smart home applications. Although some methods achieved near-perfect testing accuracy, overfitting was addressed using cross-dataset validation: models were trained on one dataset and evaluated on a fully independent dataset collected in a separate session. This ensures no temporal overlap or data leakage. The slight reduction in cross-dataset scoring confirms exposure to unseen motion patterns, providing a realistic assessment of generalization, similar to cross-subject validation. While the results show high accuracy within the collected datasets, generalization to a larger population should be considered with caution, as the total number of participants was limited and only nine daily activities were recorded. Environmental variations, sensor placement differences, and individual movement styles may also affect performance. Future work will expand the population, activity range, and real-world evaluation to improve generalizability. The consistently high-scoring accuracy demonstrates strong generalization while minimizing overfitting risk.

Besides the RF and ANN capability of capturing complex, non-linear relationships, the obtained results shows, using two complementary sensor locations (ankle and wrist) provides rich motion capture; choosing nine activities reduces ambiguity between classes, while still covering a realistic range of daily movements; the dataset is sufficiently large and well-labeled, all contributing factors to high classification accuracy obtained in this study. The application of developed models in real-time solutions allows integration with smart home systems to automatically adapt to the occupant’s activity and lifestyle, providing optimal conditions in terms of light, ambient temperature, etc. A future direction for this field could involve increasing the number of activities and further integration with smart home systems beyond lighting and climate control, such as security systems, elderly monitoring, emergency detection, etc. The high accuracy obtained in this study can be used directly to improve safety and health monitoring in smart homes. Detection of activities such as walking, climbing stairs, or relaxing, the system can identify abnormal behaviors, prolonged inactivity, or potential falls in real time. In the future, the study will expand its scope by incorporating more recognizable activities and increasing interaction with smart buildings. In addition, the gains from combining multiple algorithms, through ensemble methods or hybrid learning approaches, along with the improved robustness and accuracy it offers, will be examined and investigated.

While there have been numerous contributions to the domain of human activity recognition, this study stands out for its unique methodology, measurement techniques, integration with smart homes, and the selected activity classes. In terms of accuracy, the results match or exceed most similar previous works, and the introduction of new activity classes goes beyond the common focus on stairs, running, walking, and fall detection. Although not all daily activities are represented, the high accuracy suggests a promising direction for future expansion. The proposed human activity recognition system shows strong potential for adaptive assistance and real-time monitoring in smart homes. By identifying occupant activities, it can automatically adjust lighting, temperature, and other environmental settings, while also detecting unusual behaviors, prolonged inactivity, or possible falls. Using wrist and ankle sensors, robust classifiers such as RF and ANN, and segment-based majority voting ensures that recognition is accurate and timely, typically within 2 s. At the same time, integrating wearable HAR systems into smart homes poses challenges. Proper sensor placement is crucial, as misalignment can reduce accuracy, and variability in individual movement patterns can affect model generalization. Real-time operations require low-latency data transfer and processing, and smart home protocols demand reliable communication and interoperability. Sensor noise, missing data, and brief activity transitions can also cause misclassifications. Privacy and security are additional concerns when monitoring occupants continuously. In this study, many of these issues were mitigated by using complementary sensor locations, robust models, and majority voting, while future work will explore further improvements for real-time deployment and privacy.

To evaluate the performance of the proposed dual-location, raw-signal framework, Table 6 compares the obtained results with existing wearable HAR benchmarks. Early multi-node systems, like the widely cited benchmark by Altun and Barshan (2010), needed five separate body sensors and heavy feature engineering to reach 91.50% accuracy. Modern deep learning approaches—such as the CNN-LSTM hybrid by Mutegeki and Han (2020) and wrist-based deep models by Mekruksavanich et al. (2026)—achieve strong classification scores of 92.13% and 93.10%, respectively. However, they rely on standard single-session evaluation and overlook real-time deployment limits. Other recent work highlights similar trade-offs: Lakshmi and Poulose (2025) analyzed placement differences between wrist and ankle locations, while Miao and Chen (2026) used large foundation models to improve cross-domain adaptability (89.20%–92.40%), but these models either require high computational overhead or remain restricted to offline testing. In contrast, the proposed approach combines a targeted wrist-and-ankle placement with direct raw-tensor normalization to achieve 93.48% cross-dataset accuracy. More importantly, by exporting through PMML, the proposed system bridges the gap between high accuracy and low-latency ( <2 s). Additionally, KNX/MQTT smart home control feature is missing from current offline benchmarks.

**TABLE 6:** Comparative analysis against state-of-the-art wearable HAR frameworks.

Study	Sensor placements	Feature pipeline	Validation mode	Accuracy (%)	Real-time automation integration
Altun and Barshan (2010)	Torso, arms, legs (5 units)	Classical time/Freq features	k -fold cross-validation	91.50%	Offline evaluation only
Mutegeki and Han (2020)	Lower back, thigh, ankle	Deep CNN-LSTM	Standard train/Test split	92.13%	Offline evaluation only
Lakshmi and Poulose (2025)	Wrist vs. Ankle analysis	Statistical IMU features	Intra-session validation	91.80%	Offline evaluation only
Mekruksavanich et al. (2026)	Single wrist sensor	Deep neural networks	Standard k -fold	93.10%	Offline evaluation only
Miao and Chen (2026), Miao and Chen (2026)	Multi-node IMU array	Foundation model embeddings	Zero-shot/Cross-domain	89.20%–92.40%	High latency/Offline
Proposed work	Right wrist + right ankle	Raw normalized tensors	Cross-dataset (session)	93.48%	Live KNX/MQTT integration ( <2 s)

While the proposed wearable HAR system demonstrates high classification accuracy and low-latency smart home actuation, several limitations should be noted:

- Cohort Size and Demographic Diversity: While cross-dataset validation confirmed cross-session stability, the study evaluated a small cohort of six healthy adult male participants. The findings demonstrate proof-of-concept viability for KNX/MQTT smart home control rather than broad population generalization. Factors such as varied physical morphology, age-related gait differences, gender-specific movement biometrics, or physical impairments introduce kinematic shifts that require further testing on larger, more diverse cohorts before commercial release.
- Fixed Sensor Alignment: The classification pipeline relies on consistent positioning on the right wrist and right ankle. Physical displacement, rotation, or inversion of the wearable nodes during prolonged daily wear could induce spatial signal drift and impact accuracy.
- Semi-Structured Task Protocols: Data were recorded in continuous 5- to 10-min activity blocks. Although cross-session reliability was verified, validating the system under fully unconstrained, free-living routines remains an important next step.
- Wireless Transmission Dependency: The edge-to-host architecture requires continuous Bluetooth or Wi-Fi streaming from the body nodes. Interference or packet loss in crowded RF environments could temporarily delay window assembly and trigger latency spikes.

## 7 Conclusion

This article presented low-latency human activity recognition using wearable gadgets worn on the right wrist and ankle for real-time integration with smart home automation systems. By fusing synchronized 9-DoF kinematic streams from the right wrist and right ankle at 66.6 Hz, the system captures complementary upper and lower body movement patterns across nine daily activity classes, standing in one location with minimal movement, writing using a pen and paper, climbing down the staircase, climbing up the staircase, jogging, walking, cleaning floors using a vacuum cleaner, using a computer for web surfing, and relaxing, lying down with minimal movement. Targeting class ambiguity in complex household tasks.

The article evaluated six machine learning architectures on normalized, raw time-series windows; the results showed decision-tree models suffer from session-specific overfitting, Random Forest and Artificial Neural Network (ANN) models showed superior cross-dataset generalization, achieving 93.48% cross-session accuracy. The predictor importance analysis confirmed that vertical elevation activities, such as climbing stairs, rely primarily on lower-limb magnetometers, while fine-motor tasks, such as typing and writing, are driven by wrist accelerometers. End-to-end hardware validation confirmed that deploying PMML-exported ANN models within a C# host environment—coupled with a 120-sample majority-voting filter—enables dependable, context-aware KNX and MQTT building automation with an operational latency under 2 s.

Future work will expand the experimental cohort to include diverse demographic groups, evaluate unsupervised adaptation to mitigate sensor placement drift, and integrate edge-computing microcontrollers for direct on-device inference.

## Data Availability

The data used in this study are available in the IEEE DataPort repository at https://dx.doi.org/10.21227/08xk-r309.
